# Food limitation of sea lion pups and the decline of forage off central and southern California

**DOI:** 10.1098/rsos.150628

**Published:** 2016-03-02

**Authors:** Sam McClatchie, John Field, Andrew R. Thompson, Tim Gerrodette, Mark Lowry, Paul C. Fiedler, William Watson, Karen M. Nieto, Russell D. Vetter

**Affiliations:** 1 Fisheries Resources Division, Southwest Fisheries Science Center, NOAA Fisheries Service, 8901 La Jolla Shores Drive, La Jolla, CA 92037-1509, USA; 2 Marine Mammal and Turtle Division, Southwest Fisheries Science Center, NOAA Fisheries Service, 8901 La Jolla Shores Drive, La Jolla, CA 92037-1509, USA; 3 Fisheries Ecology Division, Southwest Fisheries Science Center, NOAA Fisheries Service, 110 Shaffer Road, Santa Cruz, CA 95062, USA; 4 Water Resources Unit, Institute for Environment and Sustainability, European Commission Joint Research Centre, Ispra, Italy

**Keywords:** sea lions, forage, food limitation, California Current System

## Abstract

California sea lions increased from approximately 50 000 to 340 000 animals in the last 40 years, and their pups are starving and stranding on beaches in southern California, raising questions about the adequacy of their food supply. We investigated whether the declining sea lion pup weight at San Miguel rookery was associated with changes in abundance and quality of sardine, anchovy, rockfish and market squid forage. In the last decade off central California, where breeding female sea lions from San Miguel rookery feed, sardine and anchovy greatly decreased in biomass, whereas market squid and rockfish abundance increased. Pup weights fell as forage food quality declined associated with changes in the relative abundances of forage species. A model explained 67% of the variance in pup weights using forage from central and southern California and 81% of the variance in pup weights using forage from the female sea lion foraging range. A shift from high to poor quality forage for breeding females results in food limitation of the pups, ultimately flooding animal rescue centres with starving sea lion pups. Our study is unusual in using a long-term, fishery-independent dataset to directly address an important consequence of forage decline on the productivity of a large marine predator. Whether forage declines are environmentally driven, are due to a combination of environmental drivers and fishing removals, or are due to density-dependent interactions between forage and sea lions is uncertain. However, declining forage abundance and quality was coherent over a large area (32.5–38° N) for a decade, suggesting that trends in forage are environmentally driven.

## 1. Introduction

A major goal of marine conservation is to protect higher trophic-level species as their removal can have dramatic consequences on entire food webs [1]. In the USA, many species of marine mammals were historically targeted by commercial or recreational fisheries [2] but with the implementation of the Marine Mammal Protection Act (MMPA) in 1972 they are now protected. California sea lions (*Zalophus californianus*) were historically heavily exploited for their oils, hides and meat from the late 1800s and were later killed by fishery interactions [3], and population sizes were low in the early 1970s. Subsequent to the implementation of the MMPA, their population increased rapidly, sustaining greater than 5% annual increase in pup numbers over the past 36 years (1975–2011) at their breeding colonies on the Channel Islands off the coast of southern California [4]. Sea lions eat a wide range of fish and squid, and their diet shows strong interannual variability, but the most common items in their diet are market squid (*Doryteuthis opalescens*), northern anchovy (*Engraulis mordax*), Pacific sardine (*Sardinops sagax*), shortbelly rockfish (*Sebastes jordani*), juvenile Pacific hake (*Merluccius productus*), Pacific mackerel (*Scomber japonicus*) and jack mackerel (*Trachurus symmetricus*) [5–9], potentially placing them in competition with fisheries. The species of fish consumed and the proportions of fish and squid in the diet differ regionally, with season and with environmental conditions [5–9]. Sea lions are opportunistic feeders that switch and increase the diversity of their diet under conditions of scarcity [9]. During El Niño conditions, their diet changes to less nutritious and less preferred species, including juvenile rockfish and hake, instead of sardine and anchovy [9]. Market squid are thought to be a less preferred species but comprise a significant fraction of the diet [7,9,10]. Melin *et al.* [9] demonstrated lower pup weights when scats from females primarily contain rockfish and squid.

**Figure 1.**
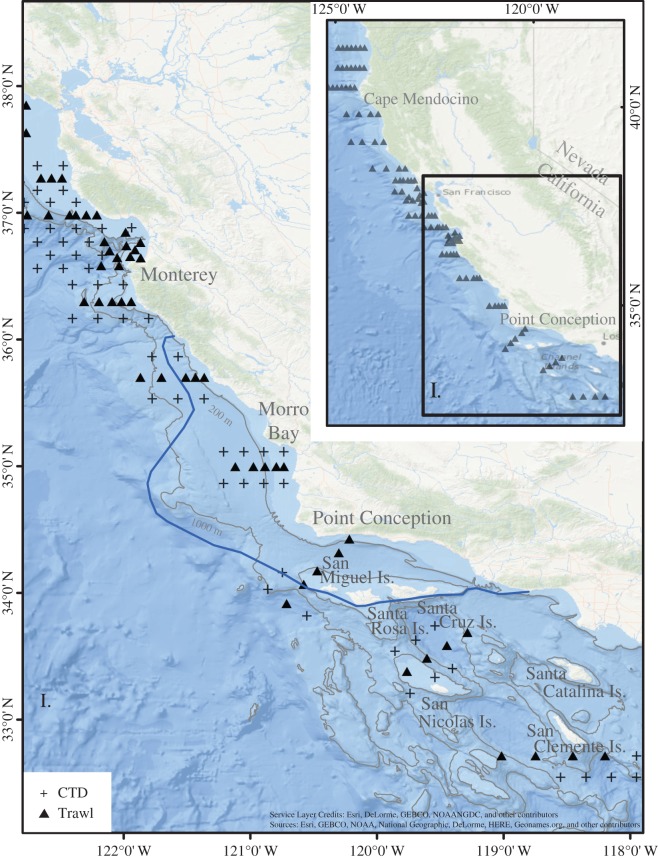
Forage sample locations and sea lion foraging range. Map of the Rockfish Recruitment and Ecosystem Assessment Survey stations off southern and central California that have been sampled continuously since 2004. Stations from Point Reyes, just north of San Francisco, to Monterey are denoted ‘central’. Stations in box I from San Diego to Monterey are denoted ‘southern’. Foraging range of six tagged breeding females in 1 year from San Miguel Island [14] in June–July are shown by the blue line. Contours at 200 and 1000 m delineate shelf and slope regions. CTD refers to locations where conductivity–temperature–depth profiles were taken.

Sea lions experience repeated years when the dependent pups are thinner than normal and show greater mortality than is usual. These mortality events have been documented in 1983, 1992–1993, 1997–1998, 2009 and 2013 (see http://www.nmfs.noaa.gov/pr/health/mmume/, last accessed 19 February 2015). The environment is believed to exert important effects, but the mechanisms are not well understood and may vary between events. Early work indicated that sea lion births and pup weights at the rookeries in the Channel Islands and within the Southern California Bight declined, and pup mortality increased, in the 1982–1983 El Niño, and took up to 6 years to recover their former level [11–13]. The effect of the 1982–1983 El Niño on sea lion births was greater at the southernmost rookeries in the Southern California Bight (Santa Barbara, San Nicolas and San Clemente Islands) compared with the northernmost rookery (San Miguel Island) (figure 1), and was associated with distinct changes in maternal sea lion diet and foraging success [11]. Recovery was slower after the 1983 El Niño than in other years, because adult females as well as pups suffered mortality [11,12]. The fact that all of the rookeries experienced higher mortality suggests environmental causative factors operating at larger than local scales. In this paper, we show that food limitation of nursing female sea lions and the consequent malnourishment of their pups is not limited to strong El Niños, but is a result of an increasing population experiencing a period of changing forage composition near the California Channel Island breeding colonies.

## 2. Material and methods

### 2.1 Study design

Relative abundances of forage fishes were obtained from the Southwest Fisheries Science Center’s Rockfish Recruitment and Ecosystem Assessment Survey (RREAS; figure 1) as log_*e*_-transformed mean standardized trawl catch rates (detailed survey methods in [15]). RREAS targets young-of-the-year rockfish and Pacific hake with a modified Cobb midwater trawl, with a target headrope depth of 30 m, but also quantifies the abundance of northern anchovy, Pacific sardine and other forage species [15,16].

There is little information available on the foraging ranges of breeding female sea lions or on the forage assemblages directly adjacent to the California Channel Islands breeding colonies (figure 1). Foraging ranges for breeding female sea lions were digitized from maps based on six tagged females from San Miguel Island in summer 1995 [14] (figure 1). To define forage trends, we selected RREAS sampling stations within the San Miguel foraging range (see blue line in figure 1) between 2004 and 2014. Data collected by RREAS prior to 2004 were from the core survey area north of Monterey Bay [15], beyond the foraging range of breeding female sea lions.

Although it is intuitive that prey items with higher fat and caloric values will be more important than those with lower fat and caloric values, precise data on which measure of food quality may be most important are unavailable. Moreover, fat and caloric content of forage vary seasonally and with body condition (e.g. depending on the presence or absence of reproductive products, or on gut fullness of prey). However, relative forage species composition, particularly with respect to higher versus lower caloric value prey, will still be an important determinant of food quality. Because we lack seasonal measures of fat and caloric content of prey near the sea lion rookery, we used species composition as a proxy for food quality when modelling pup weight. Species composition was estimated as standardized catch rates. We interpreted the results using data on the average fat and calorie content of fish and squid flesh.

### 2.2 Statistical analysis

We used linear multiple regression to determine how well prey abundance and pup sex explained pup weight between 2004 and 2014. From prior knowledge, we knew that male and female pup weights should have different intercepts. We did not consider models with only a single forage species, because sea lions are known to be opportunistic in their diet, rather than specializing on a single prey species. Based on the hypotheses stated above, and trends in the relative abundance of forage, we created two new variables to represent energy-rich forage (*sardine.anchovy*) and less energy-rich forage (*squid.rockfish*). Average food quality, in terms of the calorie and the fat content, was obtained from the NOAA Fishwatch website (www.fishwatch.gov) for fillets prepared from human consumption. The derived variables were created by adding the anti-logged standardized catch rates, and then taking the natural logarithm to create logged derived variables. Catch rates are based on individual counts rather than on biomass. *Sardine.anchovy* decrease, whereas *squid.rockfish* increase through time during the sampling period, and these variables are highly negatively correlated (*r*=0.87) with each other. No significant autocorrelation was found in either the forage variables (*sardine.anchovy* and *squid.rockfish*) or the dependent variable (*pup weight*) at any lag.

We did not include the two prey categories separately into regression models because of collinearity but instead performed a principal components analysis (PCA) on these variables using the R package vegan [17]. Principal component 1 (*PC1*) explained 94% of the total variance and correlated highly with *sardine.anchovy* (*r*=−0.94) and *squid.rockfish* (*r*=0.98) abundances.

Four candidate models were developed to explain variability in pup weight between 2004 and 2011. The full model included additive and multiplicative effects of *sex* and *prey* (*pup weight*∼*intercept*+*sex*+*prey*+*sex*×*prey*+*ε*), the additive model excluded the interaction (*pup weight*∼*intercept*+*sex*+*prey*+*ε*) and two models had only one independent variable each (*pup weight*∼*intercept*+*sex*+*ε*) and (*pup weight*∼*intercept*+*prey*+*ε*), where *pup weight* is the mean weight (kg) of male and female 14 week old sea lion pups at San Miguel Island (figure 1) rookery over each year from 2004 to 2011 obtained from [9], prey is *PC1*, *sex* is a factor to permit different slopes and intercepts for male and female pups, and *ε* is the error term. The relative plausibility of each model was characterized using Akaike’s information criterion adjusted for small sample size (AIC_*c*_) with the AICcmodavg package in R [18]. The analysis was repeated at a broad scale using forage sampled between Monterey and San Clemente Island (figure 1), and at a small scale using forage sampled from within the foraging range of female sea lions derived from published tagging results (figure 1). The broader scale analysis addressed the concern that the smaller foraging range could potentially bias the analysis if forage within that limited range was not representative of the broader region.

Out of sample model prediction for 2012–2014 was performed using predictor variables and 95% confidence intervals based on data from 2004 to 2011. The out of sample predictions were compared with pup weights in 2012 and 2013 published in Leising *et al.* [19], and to pup weights in 2014 published in Leising *et al.* [20].

## 3. Results

**Figure 2.**
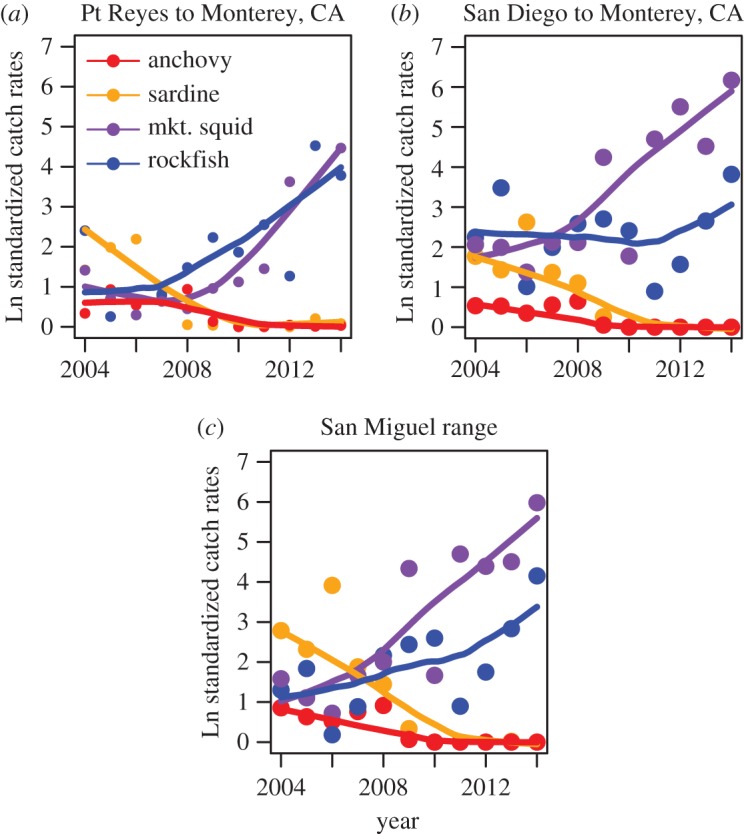
Trends in relative forage abundance. Time series from 2004 to 2014 of relative abundance of forage fish from the fishery-independent Rockfish Recruitment Ecosystem Assessment Survey (RREAS) conducted in summer off southern and central California. Relative abundance of species important in the California sea lion diet off (*a*) north central California, (*b*) south central California and southern California (see box in figure 1 for trawl station locations). (*c*) Relative forage abundance from the foraging range of breeding female sea lions from San Miguel Island rookery (see foraging range in figure 1). Smooths fitted using Locally Weighted Smoother, LOWESS [21].

During the decade 2004–2014, the forage fish eaten by sea lions showed distinct trends that affected both prey abundance and prey quality off central and southern California, and in the foraging range of the sea lions from San Miguel Island (figure 2). The abundance of aggregate forage (anchovy, sardine, hake, rockfish and squid) off both southern and central California decreased and then increased (not shown here). More important than the changes in total forage was the change in taxonomic composition of forage over this decade. The relative abundance of squid greatly increased, whereas the abundance of sardine and anchovy fell to very low numbers off central and southern California (figure 2*a*,*b*), and in the San Miguel female sea lion foraging area (figure 2*c*). Rockfish also increased, particularly on the northern central coast from Point Reyes to Monterey (Point Sur), California (figure 2*a*), but exhibited more temporal variability off southern California (from San Diego to Monterey, California; figure 2*b*). The same pattern of increasing abundance of squid and rockfish contrasting with decreasing abundance of sardine and anchovy was also evident in the foraging range of breeding female sea lions from the San Miguel rookery (figure 2*c*). These changes in forage abundance had important consequences for the food quality of forage available to breeding female sea lions at the Channel Island rookeries. The coherence of these patterns over approximately 5° of latitude shows that the shift in forage species composition is not a localized phenomenon.

The caloric densities of sardine and anchovy flesh are higher than the caloric content of market squid, rockfish or hake (table 1). Anchovy and sardine flesh is also higher in fat content compared with squid, rockfish or hake (table 1). Sardine consistently have higher calorie and fat content than other common forage taxa of sea lions. We considered that a combination of forage abundance and quality might have an effect on the condition of dependent sea lion pups. We modelled sea lion pup weights to test whether forage could be used to fit and to predict pup weights. Model selection provided overwhelming support for the additive model (weight∼prey+sex), weak support for the multiplicative model (weight∼prey+sex+prey×sex), and no support for the models with only prey or only sex (table 2). Model-averaged slopes show that the correlations between weight and prey (weight is higher when sardine/anchovy are plentiful) and weight and sex (males are heavier) are highly significant (95% confidence intervals do not intersect zero), but that the interaction term is not correlated with pup weight (table 3).

**Table 1.** Nutritional value of forage. Nutritional value of selected forage fish and squid flesh derived from fillets for human consumption (NOAA Fishwatch). Species-specific data were limited for rockfish, which are consequently presented at the generic level (*Sebastes* spp.).

taxa	cal g^−1^	total fat g^−1^
Pacific sardine (*Sardinops sagax*)	2.17	0.124
northern anchovy (*Engraulis mordax*)	1.31	0.048
rockfish	0.94	0.016
market squid (*Doryteuthis opalescens*)	0.92	0.014
Pacific hake (*Merluccius productus*)	0.90	0.013

**Table 2.** Results of four models seeking to explain variation in pup weight from 2004 to 2011 using forage sampled in the female sea lion foraging range (figure 1). Models are ordered by their relative plausibility. *Δ*AIC_*c*_ is the difference in AICc score between each model and the most plausible model. Cum. weight refers to the cumulative weight of the models, LL is the log likelihood score and *R*^2^ is the multiple *R*^2^-value for each model.

	*k*	AIC_*c*_	*Δ*AIC_*c*_	AIC_*c*_ weight	cum. weight	LL	*R*^2^
prey+sex	4	61.90	0	0.86	0.86	−25.13	0.81
prey+sex+prey×sex	5	65.55	3.65	0.14	1	−24.78	0.82
prey	3	73.35	11.45	0	1	−32.68	0.52
sex	3	79.36	17.46	0	1	−35.68	0.30

**Table 3.** Model-average results for each independent variable in the four candidate models. *β* is the slope of each term and CI stands for confidence interval.

variable	*β*	lower 95% CI	upper 95% CI
sex	2.91	1.65	4.18
prey	−3.59	−4.78	−2.41
sex×prey	−0.90	−3.31	1.50

**Figure 3.**
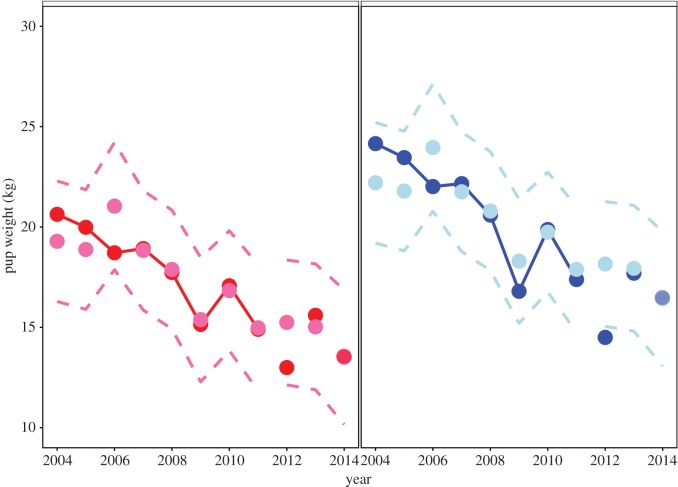
Predicted and observed trends of sea lion pup weight. Time series of observed male (dark blue), predicted male (light blue), observed female (dark red) and predicted female (hot pink) sea lion pup weights from San Miguel Island rookery obtained from Melin *et al.* [9] compared with the model predictions from the most plausible models (weight∼sex+prey) developed with data from 2004 to 2011. Dashed lines are 95% confidence intervals for the predicted values. Out of sample predictions and prediction limits are shown for 2012–2014, and compared with sea lion pup weight data for 2012 and 2013 obtained from Melin *et al.* [9] and for 2014 from Leasing *et al.* [20]. Note that the 2014 out of sample predictions overlap almost exactly with the measured pup weights published in Leasing *et al.* [20].

The best model accurately fits the observed sea lion pup weights between 2004 and 2011 (figure 3). Sea lion pup weight data always fall inside the 95% prediction intervals for the modelled sea lion pup weights for both sexes. The correlation between observed and predicted values is a simple measure to summarize the predictive power of a linear model [22]. The correlation was *r*=0.87 for observed and predicted male pup weights and *r*=0.86 for females.

Out of sample prediction was less successful in 2012 when predicted pup weights were 3.7 and 2.2 kg higher than observed pup weights for males and females, respectively (figure 3). The degree of model inaccuracy in 2012 suggests that other factors in addition to forage were important in that year. However, the model was much more successful in predicting pup weights in 2013, over predicting male pup weights by only 0.2 kg and under predicting female weights by 0.6 kg (figure 3). The out of sample model fit to pup weights in 2014 was almost perfect (figure 3), as shown by the overlaid predicted and measured data points.

The best model explained 67% of the variance using forage variables sampled between Monterey and San Clemente Island (figure 1). This area includes areas that are beyond the female sea lion foraging range determined from published tagging data. When we limited the foraging variables to areas within the female sea lion foraging range, the model explained 81% of the variance in pup weight (table 2).

There is no indication from figure 3 that the model fit was poor in El Niño years that occurred between 2004 and 2014 (i.e. in the weak El Niños of June–July–August 2004 to March–April–May 2005 and August–September–October 2006 to December–January–February 2007, or in the moderate El Niño of June–July–August 2009 to March–April–May 2010). The prey variable included in the model includes the most relevant effect of the El Niños that affects pup weights. Adding a categorical variable for El Niño would not improve the model, because the effect of El Niños on low production is already included.

## 4. Discussion

It is quite unusual to have a decade-long time series of forage fish abundance data available to match with pinniped pup weights. The Southwest Fisheries Science Center’s Rockfish Recruitment and Ecosystem Assessment Survey (RREAS) provided a unique, fishery-independent, quantitative time series of forage abundance overlapping the foraging range of breeding female sea lions. California sea lions are known to consume not only young-of-the-year rockfish and hake, but also juvenile and adult stages [23]. Among the rockfish, sea lions consume mostly shortbelly rockfish, *Sebastes jordani* [23], but other rockfish species appear in the diet when the population experiences nutritional stress. Sea lions also take advantage of strong shortbelly rockfish year-classes, consuming them for several years until they move below sea lion foraging depths [23]. Although many rockfish species are captured by RREAS, all tend to show similar temporal trends in abundance [15].

In general, there tends to be fairly strong spatial coherence in abundance in all of these forage species, although sharp differences in abundance have been noted in some years north and south of major promontories in the California Current System [16]. There is less data available regarding spatial patterns of covariation in the abundance of sardine, anchovy and squid, but there is some indication that the sea lion foraging habitat around these rookeries represents the ‘core’ habitat of northern anchovies in particular [24], such that declines in abundance in this region are likely to be reflective of broader scale declines throughout their range.

The US sea lion population increased from approximately 50 000 to 340 000 individuals (rounding to the nearest 10 000 animals) from 1975 to 2014 [25,26]. Ultimately, the increasing sea lion population would be expected to fluctuate around some as yet unknown, and probably varying, carrying capacities (depending on environmental variability) with a reduced population growth rate.

Food limitation is probably mediated through the adult nursing females’ ability to locate sufficient quality food. Unfortunately, we do not have any data to quantify how forage abundance and quality affects either the composition or quantity of mothers’ milk. Despite the relatively short time over which we have both pup weight data and appropriate forage fish data off central and southern California (2004–2014), our model results offer compelling evidence of a food limitation effect on the weight of dependent pups. The trends in the relative abundance of forage taxa and associated forage quality highlight a decade-long decline in the availability of forage for sea lions. When considered with the exponential increase in sea lion numbers, the evidence indicates food limitation of dependent sea lion pups. Food limitation, through either declines in prey density or an increase in the ratio of suboptimal prey, has been shown to contribute to observed declines in pup or juvenile survival in other pinniped populations [27–29]. For example, Trites *et al.* [30] showed that the Steller sea lion populations in the Northeast Pacific that demonstrated the greatest population declines tended to occur in the regions in which diets were the least diverse and were associated with the lowest energy prey. In this and most other examples, linkages were made between low energy, low diversity and/or declining prey abundance and subsequent predator declines, but very few studies have evaluated density-dependent impacts on food availability either in concert with or as a result of population increases. However, studies of seabirds, which are also central place foragers, have found linkages between fledging success and prey quality in instances where no relationship was evident with prey quantity (reviewed in [31], see also [32]). In one of the more unusual examples, an inverse relationship existed between abundance and predator response, owing to density-dependent impacts on prey condition [33].

Our results refocus the debate on the causes of sea lion pup weight loss from episodic stresses associated with El Niño years to a longer-term trend of declining forage quality in the waters around the California Channel Island rookeries. The large spatial scale of declines in sardine and anchovy and increases in market squid and rockfish suggests that the drivers for these forage trends are environmental. Sardine and anchovy populations both show large interannual variability that is environmentally driven [34,35] and prior to any fishing. Contrary to the paradigm that sardine and anchovy show alternating cycles of abundance, long time series show no evidence of alternating abundances [36]. During this study, sardine and anchovy were both at low abundances, exacerbating the effect of scarce high nutritional quality prey. Sardine are commercially fished and have declined in the last decade during a cool period beginning around 2000 in the California Current System. There is some indication that fishing mortality may have contributed to the declining trend in sardine abundance since 2006 [37], but this is controversial [38]. It is notable that anchovy were lightly fished in the last decade, and they have also declined [39]. The market squid fishery off southern and central California is quite large, yet squid abundance showed an increasing trend over the same period, despite the increase in landings. Rockfish are taken in both commercial and recreational fisheries, but in response to historical overfishing, allowable catches have declined, and many species increased in abundance over the period of our study. We speculate that the causes of the current forage trends are largely environmental, although the combined effects of human removals and climate are difficult to disentangle when forage populations decline as populations are fished [40].

Moreover, there are indications that the future of fisheries management will be one in which competitive interactions between human and ecosystem predators will become more tightly coupled than they are currently, with the spatial elements of such interactions being of particular importance [27]. Sea lions and the fisheries will probably share the experience of low-frequency temporal variability in forage productivity. In the near term, we expect repeated years with malnourished and starving sea lion pups, but because we cannot forecast fluctuations in forage, we cannot predict how long low forage abundance may last. Given the likelihood that the California sea lion population is approaching carrying capacity, density-dependent effects such as food limitation of pups may be a long-term consequence of a rebuilt sea lion population during the current period of low forage abundance.

## Data accessibility

Sea lion pup weight data are published in cited references. Forage data are available online through the National Oceanic and Atmospheric Administration Environmental Research Division Data Access Programme (NOAA ERDDAP; http://coastwatch.pfeg.noaa.gov/erddap/index.html).

## References

[1] Estes J *et al.* 2011 Trophic downgrading of planet earth. *Science* 333, 301–306. (doi:10.1126/science.1205106) 21764740 10.1126/science.1205106

[2] Roman J, Altman I, Dunphy-Daly M, Campbell C, Jasny M, Read A 2013 The marine mammal protection act at 40: status, recovery, and future of U.S. marine mammals. *Ann. N.Y. Acad. Sci*. 1286, 29–49. (doi:10.1111/nyas.12040) 23521536 10.1111/nyas.12040

[3] Cass V 1985 Exploitation of California sea lions, *Zalophus californianus*, prior to 1972. *Mar. Fish. Rev.* 47, 36–38.

[4] Carretta JV *et al.* 2013 U.S. Pacific marine mammal stock assessments: 2012. U.S. Department of Commerce, NOAA Technical Memorandum NMFS-SWFSC-504.

[5] Lowry MS, Oliver CW, Macky C, Wexler JB 1990 Food habits of California sea lions *Zalophus californianus* at San Clemente Island, California, 1981–86. *Fish. Bull.* 88, 509–521.

[6] Lowry MS, Stewart BS, Heath C, Yochem PK, Francis JM 1991 Seasonal and annual variability in the diet of California sea lions *Zalophus californianus* at San Nicolas Island, California, 1981–86. *Fish. Bull.* 89, 331–336.

[7] Lowry MS, Carretta JV 1999 Market squid *(Loligo opalescens*) in the diet of California sea lions (*Zalophus californianus*) in southern California (1981–1995). *California Cooper. Ocean. Fish. Invest. Rep.* 40, 196–207.

[8] Weise M, Harvey J 2008 Temporal variability in ocean climate and California sea lion diet and biomass consumption: implications for fisheries management. *Mar. Ecol. Prog. Ser.* 373, 157–172. (doi:10.3354/meps07737)

[9] Melin S, Orr A, Harris J, Laake J, Delong R 2012 California sea lions: an indicator for integrated ecosystem assessment in the California Current System. *California Cooper. Ocean. Fish. Invest. Rep.* 53, 140–152.

[10] Dyche L 1903 Notes on the food habits of California sea-lions. *Trans Kansas Acad. Sci.* 18, 179–182. (doi:10.2307/3624791)

[11] DeLong RL, Antonelis GA, Oliver CW, Stewart BS, Lowry MC, Yochem PK 1991 Effects of the 1982–83 El Niño on several population parameters and diet of California sea lions on the California Channel Islands. In *Pinnipeds and El Niño, ecological studies*, vol. 88 (eds F Trillmich, KA Ono), pp. 166–172. Berlin, Heidelberg: Springer.

[12] DeLong R, Melin S 2000 *Thirty years of pinniped research at San Miguel**Island*. In *Proc. California Islands Symposium, 29 March–1 April 1999, Santa Barbara, California*, pp. 401–406. Camarillo, CA: US Department of the Interior, Minerals Management Service.

[13] Lowry MS, Maravilla-Chavez O 2005 Recent abundance of California sea lions in western Baja California, Mexico and the United States. In *Proc. Sixth California Islands Symp., 1–3 December, 2003, Ventura, California* (eds VD Garcelon, C Schwemm), pp. 94–106. Arcata, CA: Institute for Wildlife Studies.

[14] Melin S, DeLong R 2000 At-sea distribution and diving behavior of California sea lion females from San Miguel Island, California. In *Proc. Fifth California Islands Symp.* (eds D Browne, K Mitchell, H Chaney), pp. 407–412. Costa Mesa, CA: MBC Applied Environmental Sciences.

[15] Ralston S, Sakuma K, Field J 2013 Interannual variation in pelagic juvenile rockfish (*Sebastes* spp.) abundance: going with the flow. *Fish. Oceanogr.* 22, 288–308. (doi:10.1111/fog.12022)

[16] Ralston S, Stewart I 2013 Anomalous distributions of pelagic juvenile rockfish on the U.S. West Coast in 2005 and 2006.**California Cooper. Ocean. Fish. Invest. Rep.** 54, 155–166.

[17] Oksanen J *et al.* 2011 vegan: community ecology package. R package version 2.0–2. See http://CRAN.R-project.org/package=vegan.

[18] Mazerolle MJ 2012 AICcmodavg: model selection and multimodel inference based on (Q)AIC(c). R package version 1.24. See http://CRAN.R-project.org/package=AICcmodavg.

[19] Leising AW *et al.* 2014 State of the California Current 2013–14: El Niño looming. *California Cooper. Ocean. Fish. Invest. Rep.* 55, 51–87.

[20] Leising AW *et al.* 2015 State of the California Current 2014–15: impacts of the warm-water ‘blob’. *California Cooper. Ocean. Fish. Invest. Rep.* 56, 31–68.

[21] Cleveland W 1979 Robust locally weighted regression and smoothing scatter plots. *J. Am. Stat. Assoc.* 74, 829–831. (doi:10.1080/01621459.1979.10481038)

[22] Zheng B, Agresti A 2000 Summarizing the predictive power of a generalized linear model. *Stat. Med.* 19, 1771–1781. (doi:10.1002/1097-0258(20000715)19:13<1771::AID-SIM485>3.0.CO;2-P) 10861777 10.1002/1097-0258(20000715)19:13<1771::aid-sim485>3.0.co;2-p

[23] Field J *et al.* 2007 Population dynamics of an unexploited rockfish, *Sebastes jordani*, in the California Current. In *Proc. Lowell-Wakefield Symp. Biology, Assessment and Management of North Pacific Rockfish* (eds J Heifetz, J Dicosimo, A Gharrett, M Love, V O’Connell, R Stanley), pp. 451–472. Anchorage, AL: University of Alaska Sea Grant.

[24] MacCall AD 1990 *Dynamic geography of marine fish populations*. Seattle, WA: Washington Sea Grant Program.

[25] Demaster D, Miller D, Goodman D, DeLong R, Stewart B 1982 Assessment of California sea lion fishery interactions. In *Trans. 47th North American Wildlife and Natural Resources Conference*. Washington, DC: Wildlife Management Institute.

[26] Carretta J *et al.* 2013 U.S. Pacific marine mammal stock assessments: 2012. U.S. Department of Commerce, NOAA Technical Memorandum NMFS-SWFSC-504, 378 p.

[27] DeMaster DP, Fowler CW, Perry SL, Richlen MF 2001 Predation and competition: the impact of fisheries on marine-mammal populations over the next one hundred years. *J. Mammal* 82, 641–651. (doi:10.1644/1545-1542(2001)082<0641:PACTIO>2.0.CO;2)

[28] Baker JD, Polovina J, Howell E 2007 Effect of variable oceanic productivity on the survival of an upper trophic predator, the Hawaiian monk seal *Monachus schauinslandi*. *Mar. Ecol. Prog. Ser.* 346, 277–283. (doi:10.3354/meps06968)

[29] Wolf N, Mangel M 2008 Multiple hypothesis testing and the declining population paradigm in Steller sea lions. *Ecol. Appl.* 18, 1932–1955. (doi:10.1890/07-1254.1) 19263889 10.1890/07-1254.1

[30] Trites AW *et al.* 2007 Bottom-up forcing and the decline of Steller sea lions (*Eumetopias jubatus*) in Alaska: assessing the ocean climate hypothesis. *Fish. Oceanogr.* 16, 46–67. (doi:10.1111/j.1365-2419.2006.00408.x)

[31] Österblom H, Olsson O, Blenckner T, Furness RW 2008 Junk food in marine ecosystems. *Oikos* 117, 967–977. (doi:10.1111/j.0030-1299.2008.16501.x)

[32] Kadin M, Österblom H, Hentati-Sundberg J, Olsson O 2011 Contrasting effects of food quality and quantity on a marine top predator. *Mar. Ecol. Prog. Ser.* 444, 239–249. (doi:10.3354/meps09417)

[33] Österblom H, Casini M, Olsson O, Bignert A 2006 Fish, seabirds and trophic cascades in the Baltic Sea. *Mar. Ecol. Prog. Ser.* 323, 233–238. (doi:10.3354/meps323233)

[34] Baumgartner TR, Soutar A, Ferreira-Bartrina V 1992 Reconstruction of the history of Pacific sardine and northern anchovy populations over the past two millennia from sediments of the Santa Barbara Basin, California. *California Cooper. Ocean. Fish Invest. Rep.* 33, 24–40.

[35] Skirvanek A, Hendy IL 2015 A 500 year climate catch: Pelagic fish scales and paleoproductivity in the santa Barbara Basin from the Medieval Climate anomaly to the Little Ice Age (AD 1000–1500). *Quatern. Int.* 387, 1–10. (doi:10.1016/j.quaint.2015.07.044)

[36] Field D, Chavez F, Lange C, Smith P 2011 Variations in fisheries and complex environments. In *Shifting baselines: The past and future of ocean fisheries* (eds J Jackson, K Alexander, E Sala), pp 59–76. Washington, DC: Island Press.

[37] Zwolinski JP, Demer DA 2012 A cold oceanographic regime with high exploitation rates in the Northeast Pacific forecasts a collapse of the sardine stock. *Proc. Natl Acad. Sci. USA* 109, 4175–4180. (doi:10.1073/pnas.1113806109) 22371604 10.1073/pnas.1113806109 PMC3306684

[38] MacCall AD, Hill KT, Crone P, Emmett R 2012 Weak evidence for sardine collapse. *Proc. Natl Acad. Sci. USA* 109, E1131 (doi:10.1073/pnas.1203526109) 22511724 10.1073/pnas.1203526109 PMC3358845

[39] MacCall AD, Sydeman WJ, Davison PC, Thayer JA 2016 Recent collapse of northern anchovy biomass off California. *Fish. Res.* 175, 87–94. (doi:10.1016/j.fishres.2015.11.013)

[40] Lindegren M, Checkley DM, Rouyer T, MacCall AD, Stenseth NC 2013 Climate, fishing, and fluctuations of sardine and anchovy in the California Current. *Proc. Natl Acad. Sci. USA* 110, 13 672–13 677. (doi:10.1073/pnas.1305733110) 10.1073/pnas.1305733110 PMC3746886 23836661

