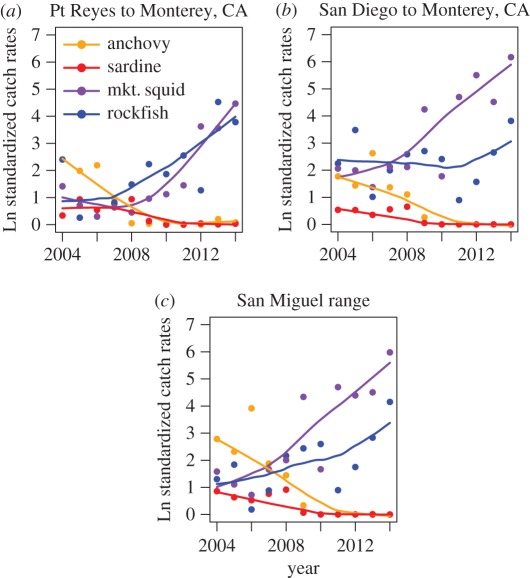# Correction to ‘Food limitation of sea lion pups and the decline of forage off central and southern California’

**DOI:** 10.1098/rsos.160192

**Published:** 2016-04-13

**Authors:** Sam McClatchie, John Field, Andrew R. Thompson, Tim Gerrodette, Mark Lowry, Paul C. Fiedler, William Watson, Karen M. Nieto, Russell D. Vetter

*R. Soc. open sci.* **3**, 150628. (Published online 2 March 2016). (doi:10.1098/rsos.150628)

This correction fixes an error in figure 2 of our published paper entitled ‘Food limitation of sea lion pups and the decline of forage off central and southern California’. The colour of the legend symbols for sardine and anchovies is reversed in the published version, but is corrected here.